# Infectious postoperative complications in oral surgery. An observational study

**DOI:** 10.4317/jced.55982

**Published:** 2020-01-01

**Authors:** Matías Dallaserra, Francisca Poblete, Cristian Vergara, Ricardo Cortés, Ignacio Araya, Nicolás Yanine, Julio Villanueva

**Affiliations:** 1 Oral & Maxillofacial Surgery Department. Cochrane Associated Center at School of Dentistry. University of Chile. Sergio Livingstone 943. 8380492. Independencia Santiago de Chile; 2 School of Dentistry. University of Chile; 3 Cochrane Associated Center at School of Dentistry. University of Chile

## Abstract

**Background:**

The main objective of this investigation was to determine the incidence of infectious postoperative complications in oral surgery.

**Material and Methods:**

A observational and descriptive study was developed, with the use of prospective registry of the patients admitted for oral surgery at the San Borja Arriarán Hospital Complex during twelve months observation period (April 2017 to March 2018).

**Results:**

The sample consisted of 532 patients undergoing surgical procedures of oral surgery and 19 cases of infectious postoperative complications. The incidence of complications reached 3,57% and it was observed predominantly in exodontic type interventions. The most commonly observed complication was dry socket, reaching 2,5% of third molar surgeries and 3,7% of extractions of other teeth. Other postoperative complication were 7 cases of facial spaces abscesses, also observed predominantly in exodontic type interventions.

**Conclusions:**

The results were similar to those reported in the literature both in their frequency and in the type of complication.

** Key words:**Oral surgery, incidence, postoperative complications, dry socket, third molar.

## Introduction

Extraction is one of the most performed procedures in oral surgery and its objective is to remove affected teeth with any pathology that compromises the health of the mouth, with third molars being the most frequent (1). The extraction can be a simple or complex procedure and this will depend on the factors that affect its removal (2-3).

In the case of the lower teeth, the thickness of the mandibular cortex compared to the maxilla and the proximity to the inferior alveolar nerve, if the tooth is included, erupted or semi erupted and the need to make a flap, osteotomy and / or odontosection, the degree of impaction, the age of the patient, experience of the surgeon and time of surgery and the anatomical considerations of the tooth (2,3).

The complications that may occur after a tooth extraction correspond to the alveolitis, hemorrhage, wound dehiscence, fracture of the bone cortices, among others (4). Despite being a routine procedure, patients have reported complications ranging from 1% to 30.9% (5-9). The most frequent post-extraction complication is alveolitis (10-15) and occurs with a frequency that varies from 0 to 35% of all dental extractions (5). Other commonly reported complications are paresthesia of the mandibular nerve, pain and infections (3,16,17).

The main objective of this study was to determine the incidence of infectious complications after oral surgery in patients over 12 years of age treated at the San Borja Arriarán Hospital in Santiago de Chile. The secondary objectives correspond to performing a descriptive analysis in relation to the characteristics of the patients included, describing the rate of complications according to the procedure of oral surgery performed and describing the different types of complications and the incidence according to the type of procedure. This study was elaborated using the STROBE statement guideline (18).

## Material and Methods

This study corresponds to a descriptive and exploratory study that expects to report the frequency and distribution of the event of interest (infectious complications after oral surgery procedures) in a highly complex urban center, in the Maxillofacial Surgery Unit of the San Borja Arriarán Hospital Complex, in Santiago de Chile from the registration of the complete casuistry of twelve months of study (April 2017 to March 2018). To carry out this research, the approval of the Ethics Committee of the Central Metropolitan Health Service was previously obtained. All the Dentists of the Unit participated.

All patients with indication of oral surgery were selected, who approved their participation in the study through an informed consent and who fulfilled all the selection criteria defined below:

• Inclusion criteria: Patients over 12 years old, systemically healthy or with controlled pathologies.

• Exclusion criteria: Patients with allergies or contraindications to the use of paracetamol or nonsteroidal antiinflammatory drugs (postoperative medication of first choice in oral surgery, the use of other alternatives could create confusing factors), who have undergone antibiotic treatment for at least 30 days before surgery (for possible residual effects of the antibiotic that modify the normal microbiota and decrease the probability of infectious complications), history of pericoronaritis up to 7 days before the intervention, patients with psychiatric illnesses that require special procedures, such as sedation or anesthesia , immunocompromised (due to their inherent higher risk of complications, especially infectious) and those who did not attend the control appointment or who did not answer control calls.

All surgeries were performed with surgical instruments properly sterilized and complying with the health requirements of the Ministry of Health. Surgeons performed surgical hand washing for 4 minutes with 2% chlorhexidine gluconate gel soap, and used sterile gloves. The surgeries were performed under locoregional anesthesia. The postoperative indications were delivered and explained both orally and in writing. Operators were recommended to use one gram of paracetamol every eight hours for three days in simple procedures and add another non-steroidal anti-inflammatory for moderate or complex procedures.

The patient was referred to control at 7 days to be evaluated and called by phone at 30 days. In case of complications, they were treated at the time the patient consulted. The clinical parameters were evaluated by the data collector who was a trained dental surgeon in the diagnoses of complications. These data were recorded in the data registration form prepared for this study.

The presence or absence of postoperative complications was considered. To determine infectious complications, the criteria for the CDC (Center for Disease Control and Prevention) classification for nosocomial infection of the oral cavity were used.

The presence of alveolitis was defined in the clinical examination when disintegration or absence of the clot associated with moderate or intense pain was observed (about 4 on the analogue visual scale, from 0 to 10) after 48 hours of the intervention.

The independent variables were considered with respect to the patient as age, sex, systemic diseases, smoking habit and alcohol or marijuana consumption, to the operator and his expertise as a dental surgeon, resident of the specialty of maxillofacial surgery or maxillofacial surgeon, surgical time (0 to 15 minutes, 16 to 30 minutes and 31 and more minutes) and surgery variables such as surgical difficulty according to whether it was mild, moderate or high. Defining as moderate those in which it was necessary to perform a flap and high difficulty procedures in which osteotomy and / or odontosection was also performed. It was also considered if there was use of antibiotic medication as antibiotic prophylaxis or later antibiotic therapy by dental indication.

Statistical analysis: A descriptive analysis of the information was carried out. The incidence of complications after oral surgery was calculated in a general manner, by type of procedure and according to the type of complication, using rates. Complications in their totality as a numerator and total procedures as denominator were considered for the general calculation. For the calculation of incidence according to type of procedure, the numerator was the associated complications for each of them and the denominator was the total of procedures of each type. All analyzes were performed using the Stata / SE version 15.0 program.

## Results

During the twelve months of the study, 682 oral surgery procedures were performed, of which a total of 106 did not meet the selection criteria and 44 were patients who did not attend the control appointment and did not answer the telephone calls. These patients were excluded from the study and the total number analyzed reached 532 patients undergoing oral surgery of which 67% were women, 35% were patients with some basic pathology and 74% had some type of habit of consumption (alcohol, tobacco or marijuana). The average age was 30.4 years with a standard deviation of 17 and 66% of the patients had already undergone some procedure of oral surgery previously.

The most performed procedure were third molar extractions, reaching 68% (n=363) of the total surgeries performed. All the complications occurred postoperatively and were mainly concentrated in the procedures of type exodontia.

Of the patients included in the study, 357 correspond to women, of which 13 presented complications that represent 2.4% of the total. On the other hand, 175 patients correspond to men of whom 6 presented complications, which represent 1.1% of the total. Regarding the medical history of the patients included, 188 presented systemic antecedents, of which 4 presented complications representing 0.75% of the total. On the other hand, 350 patients presented a history of previous oral surgery, of which 11 presented complications representing 2.06% of the total. With respect to the habits of the patients included, 132 consumed tobacco of which 4 presented complications, representing 0.75% of the total. Alcohol consumption occurred in 207 patients, of which 6 had complications, representing 1.12% of the total.  Table 1 details the characteristics of the patients included and their incidence of postsurgical complications.

**Table 1:**
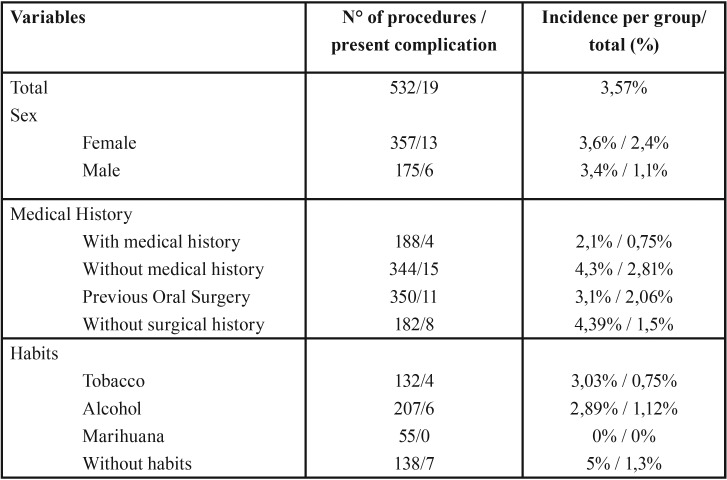
Characteristics of patients who presented complications.

The most frequent oral surgery procedure that presented the most complications was the extraction of third molars. Of the 363 patients undergoing this type of surgery, 16 presented complications, which corresponds to an incidence of 4.4% for this procedure. While the extraction of other teeth presented an incidence of 3.7%. Patients undergoing other surgeries that were considered in this study did not present complications.  Table 2 details the percentage of incidence for each type of procedure in oral surgery.

**Table 2:**
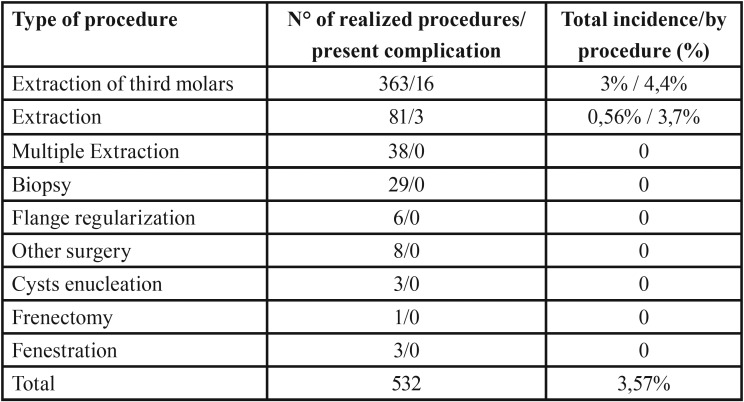
Complications by type of procedure in oral surgery.

The most frequent complication after oral surgery corresponds to the dry socket, which developed in 9 patients who underwent third molar surgery (incidence of 2.47%) and in 3 patients who underwent extraction of other teeth (3.7% incidence). The other complication that arose was the abscess of facial spaces that only occurred in patients who underwent third molar surgery (incidence of 1.92%). Types of complications observed are detailed in T Table 3.

**Table 3:**
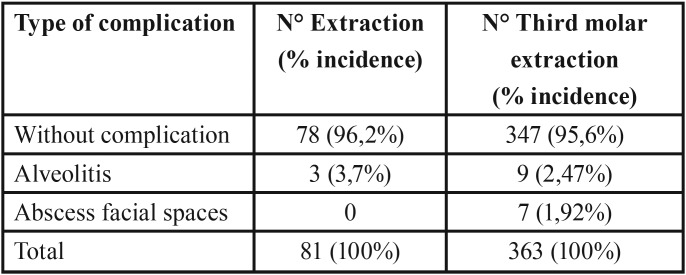
Types of postsurgical complications and their incidence according to surgical procedure.

Regarding the operator-dependent factors, the category of resident of the maxillofacial surgery specialty presented the highest level of incidence of complications after oral surgery (5.29%), followed by the category of maxillofacial surgeon (3.3 %) and dentist surgeon (1.85%). On the other hand, the surgical time interval that presented the most complications was surgeries that took more than 30 minutes (5.2%), followed by the interval of 16-30 (3.44%) and 0-15 minutes (3 , 08%).

With respect to the factors depending on the procedure, the surgical difficulty that presented the highest incidence of complications after oral surgery was severe (5.8%), followed by mild (3%) and moderate (0.7%). Finally, patients who received prescription antibiotic prophylaxis had an incidence of 4.4% of complications after oral surgery.  Table 4 contains the characteristics of the surgeries and the operator in the group that presented complications.

**Table 4:**
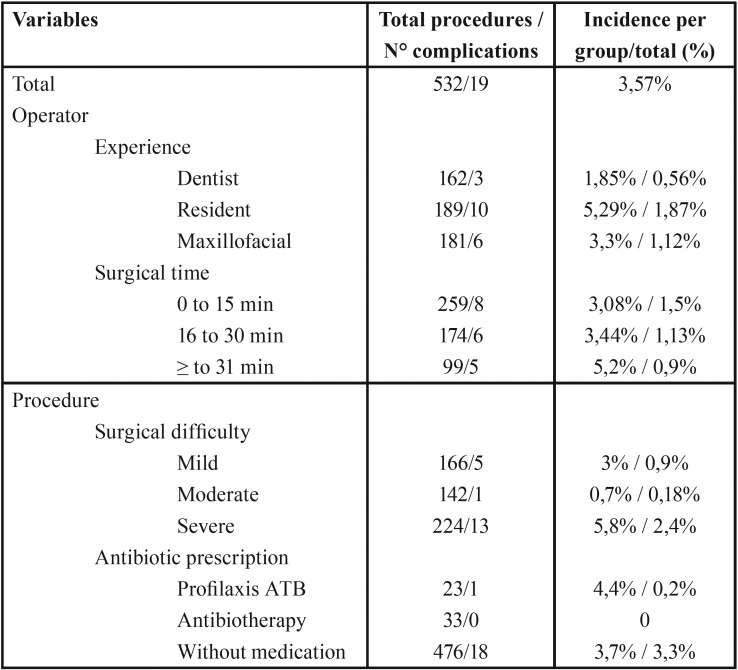
Complications by type of procedure in oral surgery.

## Discussion

The incidence of complications in oral surgery of this study reached 3,57%, mainly observed in procedures of exodontia and postoperatively. The highest percentage of complications was observed in the extractions of third molars, in 3% of the total procedures and 4,4% of the total of surgeries of this type. Most of these complications were alveolitis, reaching an incidence of 2.47% for third molar surgery. The observed result is within the wide range existing in the reports of the referring literature, since these fluctuate between 1% to 30% (5-9). Although there is no documentation of other studies equivalent to the present, it is possible to make comparisons with research whose methodologies and objectives are similar. The difference with respect to the total occurrence of complications may be due to the definition that exists about these events, since, for these authors, edema, pain and trismus are considered postsurgical complications, however, in this study, these situations were considered expected within the context of an inflammatory process that is inevitable after oral surgery.

A study with results similar to those described in this study is carried out by Bui (9) who, with 583 patients undergoing third molar extraction surgery, obtained a total of 4.6% of complications and 3.4% of alveolitis. In a favorable way, we can compare the results with the study by Eshghpour and Nejat (19) with a sample of 256 surgical procedures for extraction of impacted third molars where they observed a prevalence that reached 19.14% of alveolitis. However, this favorable difference could be due to the complexity of the procedures included, since in the cited study only impacted third molars were contemplated, however, the design of this study included surgeries with equivalent surgical complexity levels and also of lower degree.

Another similar prospective study was carried out by Chuang (20) with a total of 4,004 patients undergoing third molar extraction surgery who observed a prevalence of complications of 18.3% and 7.4% in the case of alveolitis.

When we consider the incidence of alveolitis in isolation to the other postsurgical complications, it is observed that the procedure with greater frequency of this event is tooth extractions other than third molars. For this type of intervention, the incidence reached 3.7%. It should be noted that this type of procedure was mostly performed by operators with less experience in the service analyzed, who were dental surgeons. Halabi (11) maintain the influence of the operator’s experience in the development of alveolitis, since those operators with greater practice would have more elaborate techniques and carried out in shorter times.

Cases of abscesses of facial spaces reached 1.9% of the total number of third molar procedures, a result slightly higher than that obtained in other studies, such as the one conducted by Bui (12) who, with 583 patients undergoing extraction surgery third molars, observed postoperative infection in 0.8% of cases. However, Kaczmarzyk (21) have described that the occurrence of this outcome ranges from 1 to 15%. This difference and wide range may be due to the free definition used in the different studies.

As a virtue in this study we can highlight the prospective type design, which is beneficial, since it decreases memory biases. As limitations, we can mention the inequiTable distribution in terms of the types of procedures, considering that the great majority of these were extractions makes it difficult to obtain conclusive results regarding the incidence of postoperative complications in other types of oral surgery interventions.

The results obtained show an incidence of postoperative infectious complications in the San Borja Arriarán Clinical Hospital in patients older than 12 years of 3,57%, being similar to that reported in the literature.
